# The Nursing Supplement

**Published:** 1889-10-26

**Authors:** 


### The Nursing Mirror.

All Contributions for this Supplement should be addressed to the Editor, The Hospital, 139-140, Salisbury Court, Fleet Street, London, E.C., and should have the word "Nursing" plainly written in left-hand top corner of the envelope

### En Passant.

THE PENSION FUND.—Several facts of interest to nurses were not included in the business report of the Pension Fund we printed last week. For instance, the fact that grateful letters by the dozen reach the office, from those nurses who are already profiting by the scheme. Again, some half-dozen nurses have made spontaneous contributions to the bonus fund; one sends 10s., another sends 1s.; just what they can spare they send, and it is a great proof of how thoroughly appreciated are all the blessings of the fund.

CHRISTMAS DECORATIONS.—Already queries are coming in respecting ward decorations for Christmas; in the busy life of a hospital ward, it is necessary to take time by the forelock. A very simple form of decoration consists of holly wreaths made of leaves only. The leaves are cut off singly, without stalks, and threaded through the middle on coarse thread. The prickles keep the leaves slightly apart, and they make a wreath which will twine gracefully round pillars or festoon from bed to bed. One semi-convalescent patient can clip the leaves off the boughs, and another can thread them. Yards and yards of this wreathing can be made in a very short time, and it is easy to put up, and keeps tidy for days.

SHORT ITEMS.—Mr. Laurence Humphry, of Addenbrooke's, is going to add another to the already numerous textbooks of nursing.—The enquiry into the death of Ellen Noris at Whitworth Hospital has resulted in the nurses being declared competent and kind, and free from all blame.—Miss Motibai R. Kapadia is doctor in charge of the Victoria Jubilee Dispensary for Women and Children at Ahmedabad.—A daughter of Sir Morell Mackenzie has written a paper on "Women and Athletics" for the *New York Sun.*—The five nursing sisters at the Greek Hospital, Alexandria, are English, but the servants under them are Greek or Arab.—It is proposed to endow a bed in the new Hospital for Women in memory of Miss Caroline Ashurst Biggs.—Miss Lee has been thanked for acting as matron for a month to Dublin Eye and Ear Infirmary.

NURSES AND TEMPERANCE.—The modern nurse has to live down the unenviable reputation of the old style of nurse, and hence it is that temperance principles are recommended now to all nurses. Nearly every institution makes it a rule that its nurses shall only take stimulants under doctors' orders, and if some of us find the rule irksome at times, we must remember it is enforced to secure a better reputation to our profession generally. Even now district nurses (and others) have sometimes to be dismissed for intemperance, and so-called midwives will boast of their powers of drink. The temptation to a nurse to take to stimulants is very great; shame and opportunity denying her spirits or wine, she will often take to morphia or other soothing drugs. At the Belfast Home for Nurses there used to be—perhaps still is—an excellent temperance union for nurses, of which Miss A. T. Bristow was secretary. It commenced in 1882 when there were about 50 nurses in the home, and 15 probationers were trained every year; thus the union became a strong one. A monthly letter was sent to each nurse, and, by the common promise and common prayer the bond of fellowship was kept very close. All this shows how great is the change in the nursing world since the days of Sairey Gamp.

TROUBLESOME PROBATIONERS.—There is a class of girls, unfortunately known to most matrons, who are imbued with the idea that they are born nurses, and who are always entering hospitals as probationers. When at the end of the trial month these self-satisfied damsels are dismissed, they become insolent, and subsequently write threatening letters. One would-be nurse has treated three matrons thus, and we would therefore warn other matrons to carefully enquire whether anyone applying for the post of probationer has had experience elsewhere, and if so, to write in confidence to her fellow-matron, and ask her opinion of the would-be probationer. Matrons must be frank with one another, and their letters can always be marked "private" when they refer to the character of a probationer. As a rule, an asylum superintendent refuses to take an attendant from any other institution. This is rather a drastic remedy for ridding oneself of discontented, unteachable probationers; but certainly a probationer should never be taken from another hospital unless the reason of her leaving is known.

THE HOPE INFIRMARY.—It seems that the story of the rats in the matron's bed is only the final one of a long list of scandals at Hope Hospital, which during the eight years of its existence has had five matrons and two Government enquiries. The first matron came from the Salford Workhouse with the first batch of patients, but she was shortly replaced by Miss Bloomer, who was matron for about four years, and during her reign things went com paratively well. Another matron was Miss Read, but she found her position too uncomfortable to be long retained. Seen by itself the practical joke of rats in the bed seemed a small thing, but seen in the light of a long list of outrages with which we are now familiar, we can well understand and appreciate the stern wrath of the guardians. At the last meeting of the Infirmary Committee it was resolved on the motion of Monsignor Gadd that the following report be made to the board: "Letters were received from all the nurses and servants of the infirmary in reply to the clerk's letter of October 11 denying that they had any knowledge of the occurrence referred to therein. Information, however, contained in the said letters led the committee, after careful investigation, to discover that one of the patients in the imbecile wards had placed the rats in the late matron's bed out of a spirit of revenge, or in retaliation for some imaginary wrong. The committee therefore recommend that the notice given to the nurses and servants be withdrawn." Councillor Ashton said he would move a resolution which he thought would be acceptable to the board, having regard to all they had heard of this matter. He was sure that those members who knew anything of the proverbs would remember what one of them said concerning an unruly tongue. He thought that all this unpleasantness had arisen from that source. He did not believe that any of them wished to do an injustice to a single person. They had now arrived at a stage when they should exercise mercy and consideration, and to settle the question he would move the following resolution: "That the board having learned that the insult offered to the late matron was the work of a maniac, hereby withdraws and regrets that it issued a threat of dismissal to the staff of nurses and servants at Hope, and expresses sincere sympathy with and confidence in them in the future." The resolution should also include the officers because a reflection had been cast upon them. The resolution was not seconded, and therefore lapsed.

### Cottage Hospital Matrons.

### II.—THEIR NURSING DUTIES.

A cottage hospital matron must, above all things, be a "trained nurse" that she may be able to superintend and train her probationers. To the ordinary knowledge expected of every trained nurse, which need not be specified here, she must add in many ways that of the work expected of dressers and junior house surgeons, a training really given in many large provincial hospitals to nurses. Thus she must be able, if required, to test urine chemically, take down prescriptions, dispense drugs, give hypodermic injections, pass catheters, stop hæmorrhage in emergencies until a doctor can come, put on plaster of Paris and other appliances, and extension apparatus, prepare the theatre for every class of operation. She should understand the measures taken to recover from various forms of collapse, how to watch a patient under an anæsthetic, the care needed for keeping instruments and drugs in good order, the best methods of taking in accidents, and the sort of bed required in each case. It is essential to satisfactory working of this class of hospital that the matron can, if required, do all these things, and this will in no way trench on the province of the medical officer; but as a matron, writing for "a would-be matron," I would advise that she be ready to do any or all of these things if asked, or she finds it expected from her, but not presume to offer any work beyond the plain duty of a "trained nurse." The matron will probably have the care of the daily case book, entering names, diseases, operations done, dressings, diet and medicines ordered, and will have to find out the previous history of her patients. In training probationers she will do well to interest and instruct them in the choice of suitable books to read, explaining, as far as she knows, the reason of every order given, encouraging them in adding to the ordinary care of their patients an interest in making things bright and cheerful; keeping the wards pretty with pictures, flowers, plants, and ornaments; reading and singing to them, showing interest in their pleasures, and sympathy with their pains. All this is possible in hospitals where, between the heavy busy days, comes many an easy hour. Of course they must learn to make pads, slings, prepare all sorts of dressings, besides the ordinary work of every hospital nurse. Thus, in the first instance, having trained her nurses, she will find that they work in accord with their medical officers and her own wishes, in time, I should hope, becoming cottage hospital matrons in their turn. She will do well, later on, to let them see the household working in its various forms. Or this training should equally fit them for charge of wards in the provincial hospitals, thus fulfilling Mr. Burdett's suggestion that cottage hospitals should work as feeders to their own county or mother hospital.

(*To be continued.*)

### In Loving Memory

(of E. F., late Night Sister at Manchester Infirmary).

She walked the lonely corridors in the silent dead of night,
All else was dark and dreary, but she was ever bright.

She never thought of self when work or duty must be done,
She always tried her very best to comfort everyone.

There was no parade of what she did, she did it in a cloud;
But clouds would break, and through the wards her praises echoed loud.

We have lost her, we have lost her! But our loss to her is gain;
Yet our lonely hearts will not be still, they call her name again.

She stood beside a little child to win it back to health;
They both are gone; ours, ours the loss, and theirs the heavenly wealth.

A little while to work and wait, and then the glorious end,
'Midst angel hosts our hands shall meet, our voices once more blend.

Nurse L. B.

### An Exhibition of Nursing Uniforms.

### FINAL PARTICULARS.

The following are the rules for those competing in the Doll Show: The dolls must not be shorter than four inches nor taller than twelve inches, and must represent the sister, charge-nurse, or probationer of the hospital. Any nurse intending to compete must send her name and address on a post-card, headed "Doll Competition," together with the name of the institution or hospital, the uniform of which is to be faithfully reproduced, to the Editor of The Hospital, The Lodge, Porchester-square, London, W., before next Thursday, the 31st inst. Dolls will be received at The Lodge, Porchester-square, London, W., between Nov. 24 and 30, but not before the first date. A committee of three, consisting of two matrons of hospitals and the Editor, will award £10 in prizes as they shall see fit. To each doll must be pinned a paper stating what hospital and what grade the uniform represents, and also the full name and address of the dresser. Since the list published in our pages on October 5, nurses from the following institutions have joined: Salop Infirmary, Swindon Victoria Hospital, Liverpool Children's Infirmary, Metropolitan Hospital, Devonport Albert Hospital, Brighton Nursing Association, and Liverpool Northern Infirmary. But we still desire competitors from Manchester Royal, Aberdeen, Charing Cross, Gloucester, Bristol, Exeter, Macclesfield, Hereford, Hertford, Bootle Borough, Jenny Lind Infirmary, St. John the Divine, Nottingham, Addenbrooke's, Jaffray Hospital, Worcester, Bradford, Scarborough, and many district nursing institutions. And where are our friends from Middlesex and Great Ormond-street? We have got a delightful list of names, but it would be so much more interesting to the whole nursing profession if the exhibition were complete.

### Everybody's Opinion.

[*Correspondence on all subjects is invited, but we cannot in any way be responsible for the opinions expressed by our correspondents. No communications can be entertained if the name and address of the correspondent is not given, or unless one side of the paper only be written on.*]

### SUPERANNUATED AT FORTY-THREE.

One Who Is Forty-Three writes: Being myself over 40, I should like to give a word of sympathy to the two nurses who have made their cases known to The Hospital. It is a puzzle to myself and many others why, in the nursing world, experience seems to count for nothing. I know the comfort it has been in many cases when an older, more experienced nurse is sent to a private case, and the annoyance in others when a young, inexperienced woman has arrived to nurse a critical case. It appears to me that every year a nurse worthy of the name must gain additional valuable experience. A doctor is preferred because of experience, but a nurse, in these days, is promoted because she is young, good looking, or possesses influential friends. A nurse's greatest crime seems to be to have attained the age of 40, when one would imagine she must be better fitted for positions of responsibility and authority. I have often wondered what is a nurse to do between 40 and 60 if, unhappily, she should be thrown out of employment? I should be glad to know the opinion of others on this very important subject to so many who have devoted many years to the sick and suffering. In the outside world a woman of 40 is not set on one side as too old for work, then why in the nursing world?

### Notices.

Mr. C. Alan Palmer, of 29, Ludgate-hill, is now arranging classes for men and women in connection with the St. John Ambulance Association. The men's class will be held at St. John's Gate, commencing October 29th, at 8 p.m. The ladies' class will commence next month.

Dr. F. R. Walters is giving a course of ten lectures on "The Laws of Health," at Essex Hall, on Thursdays, at 8 p.m. Fee for the course, 2s. 6d.

The London Young Women's Christian Association (offices, 316, Regent-street) are getting up some ambulance and nursing lectures for the coming winter.

The winter session of the Midwives' Institute and Trained Nurses' Club, 15, Buckingham-street, Strand, commences on Nov. 1st, when Dr. G. H. Savage will deliver a lecture, at 7.45, on "Early Symptoms of Mental Disorder after Confinement, and their Treatment." Admission to non-members, 6d.

### The Two Surgeons.

Mr. Newland (or Dr. Newland, as he was usually called for the sake of courtesy) was a country surgeon, practising in and about a pretty but decayed old market town, which went by the name of Dunstone. He lived in a quiet, unambitious sort of way near the centre of the town, and in a gabled, old-fashioned house which was remarkable for its appearance of rustic beauty and gentility. His housekeeper and companion was an only daughter whom he called Grace, and who was withal a very promising young lady, whose appearance and ways very well corresponded with her name. Dr. Newland had been a widower for many years, but his daughter was now grown up to the age of nineteen years, and was to her father in many ways what her mother could no longer be. He was a short, portly old gentleman, somewhat inclined to obesity and, in his own way, very dignified and aristocratic. He was very jealous of his reputation as a medical man, and prided himself on his superior attainments, which, to tell the truth, did not stand a great number of chances of being put to any very severe test. The reason of this was that his practice consisted chiefly of a sort of general round to the families of the neighbouring gentry, where his time was spent in administering to those little faddling indispositions which some weak female souls often think it elegant to possess. He was, generally speaking, a very good-tempered doctor, and very entertaining in his conversation, qualities which in many cases form a great requisition. Of course, all this was so long as things went well and evenly with him. When they were otherwise, he was what is generally termed "crusty." Now, as it happened, things *had* gone well with him for many years. He had been doing well, he had been well respected, and was so still, and he had been watching from day to day the growth of a promising daughter with true fatherly pride. But there had recently come a little change. There was something that had occurred lately which had crossed him considerably, and made him a trifle (nay, more than a trifle) ill-tempered—ill-tempered sometimes even with his daughter, Grace, and that was a very unusual thing.

The fact of the matter was that someone had had the impudence and audacity to bring medical credentials and to set up in Dunstone in unblushing opposition to him—him, the reputable and sole doctor of the district for I don't know how many years. This circumstance had troubled him a good deal. Not that he, to quote his own words, objected or could object to anyone making a competency for himself, but it was the—the impudence of the thing altogether that he couldn't get over.

The object of the old gentleman's spleen was a young doctor, who had recently settled down in Dunstone with the hope of securing, in a short time, a practice sufficient in its pecuniary returns to support himself and his aged mother, who was dependent upon him. In a way, the two doctors presented a strange contrast—the one was free from any anxiety with regard to means, was comfortably situated, well respected, and was in every way happy in his domestic affairs; the other had his way to make, under poor and adverse circumstances, and in the exercising of an anxious filial duty: the one case presented rather the playful side of life, the other the anxious and pathetic. Lionel Wentworth, the new surgeon, had been in Dunstone for some five or six weeks, but, as yet, the cloud of despondency hung over his prospects, for it is remarkable how difficult it is to overcome prejudices, which, in such cases, always go against a beginner; and prejudices were well abroad. First of all, Wentworth was young; then, again, his ability was almost altogether untried and unknown, and, worst of all, he was poor, and gave every evidence of being so. All these things considered and weighed, it is not to be wondered at that the young doctor was already making proposals to his mother to remove to some other part of the country, where his abilities might stand a better chance of unprejudiced recognition, and where his services, therefore, would be in better demand.

But, in spite of all this neglect of the new comer on the part of the worthies of Dunstone, there was one who had noticed him and, having seen him once, had seen him (either by chance or good management, whichever you will) many times and oft. Not Dr. Newland! He had always purposely avoided even seeing him—had absolutely denied himself the gratification of his curiosity to even cast a look when near to him, and when he could do it unobserved. No, but the person into whose good graces the young doctor had worked himself was no other than that great worthy's only daughter, Grace Newland. That young lady had by chance noticed Wentworth as soon as ever he had come into the district. The circumstance of her first introduction to him was over a very trifling affair, and, in fact, one that hardly bears recording (so trifling it is) but for the events which afterwards transpired. She had been wandering about the woods which skirted the town of Dunstone in search of flowers, and had got her light summer dress entangled in a quantity of brambles. Just at that moment Wentworth had appeared upon the scene, and had, with that gallantry belonging more to old English fashion than to the present day, gone to her assistance and succeeded in extricating her from her predicament; making at the same time a few well-chosen remarks calculated to relieve her embarrassment. Grace did not know then that he was her father's competitor in the medical field. And, in fact, when she was afterwards aware of it, it did not deduct one atom from the good opinion she had immediately formed of him; a good opinion which had in it a large share of admiration, and which was rapidly ripening into something more ethereal in its composite parts. Had Wentworth been as light of soul, and as free from anxiety and perplexity of thought as she was, it is most probable that the same state of things would have been at work in his own mind, but further than a transient corresponding feeling at the time, the thought of her was lost in the greater proportions of his weighty troubles.

Things had been going on in this way for some months, Dr. Newland grumbling a good deal about the young man of whom he knew nothing, and Grace taking every available opportunity of seeing him in order that she might know something, when a circumstance occurred which entirely altered the whole aspect of affairs as they had existed so far. The elder doctor had come down one morning in a state of great irritability. It was a rule with him on such occasions to address all his complaints to Grace, and a little battle of words (which was in reality of no consequence at all, and which, to a third party, would have appeared more playful than anything else) usually took place between father and daughter. Grace always knew how far she might venture with him, and she also knew that in certain respects she possessed a sort of power over him, that power consisting in an ability to get the better of an argument by winning him over to a better frame of mind. It was usual with the dortor when in those fits to find fault with everybody and with everything, and just now that young Wentworth was harassing his mind, he naturally vented a little of his abuse on the fortunately unconscious head of that young man.

"A doctor, indeed; a mere boy without even an apology for a beard——"

"But, papa," said Grace, "how do you know whether he has a beard or not, when you say you never look at him?"

That was a good start for Grace, and her father saw it and was pleased with her repartee, though he did not own it.

"Well, nor I have, but that is what I should judge from ——"

"But, papa, you know it is not kind, nor is it right, to judge of people we do not know merely from what we hear said."

"Well, well, perhaps I don't know—perhaps I don't know anything at all about him, nor about anybody, nor anything. Perhaps you know?"

Grace was about to say that she did know, but she remembered herself and stopped.

"And to come to a place like Dunstone," the doctor went on, "where one can do all that needs doing within a dozen miles round, at a snail's gallop, and find time afterwards to twiddle his thumbs."

Just then their conversation was interrupted by a hearty ring of the surgery bell, and Grace went out to know its purport from the servant, leaving the doctor grumbling away with poor Wentworth as his unfortunate victim.

*(To be continued.)*

### Death in Our Ranks.

On the 9th instant, deeply regretted, died Alice Guite, daughter of Mr. James Guite, Pass House, Pittsmore, and nurse at St. George's Home, Sheffield, aged 27 years. She died of typhoid contracted during her attendance on a patient whom she nursed with such care and devotion, that she was in a great measure the means of saving her life. She was buried at Burngreave Cemetery on the 12th instant, several of her fellow-nurses in uniform following her to the grave. The coffin was covered with wreaths sent by the superintendent and nurses from St. George's Home, Mr. and Mrs. W. Mountain, Mr. and Mrs. Griffiths, and other friends.

### Our Nurse.

Honour to the noble will
And the brave endeavour !
Heaven bless her healing skill
To thy use forever ;
Soothing touch and kindly smile,
Comfort sweetly spoken,
Make the spectres flee, awhile,
For the shades are broken.

Be the ward where light is dim
Lit with Love's own essence ;
Where the fuller glories swim
Breathes no fairer presence.
And the wan smile breaking o'er
Lips that pain hath tightened,
Shall reflect the far "before"
God's own hand hath brightened.

And, perchance, thine eyes will see
Many a sad existence
Pass into eternity,
Out—beyond Time's distance.
"Come to Me," a voice hath said,
Sorrow blooms in beauty,
May the Lord of quick and dead
Cheer thy tender duty.

So, God-speed !
Our tempting gains—
Fame and wealth—are hollow ;
Unto to all a "rest" remains,
And the "good works" follow.
Be the deathless blessing thine :
Christ-like the endeavour :
May His "light of healing" shine
On thy path forever !

Emily Hughes.

### Notes and Queries.

To Correspondents.—1. Questions may be written on post-cards. 2. Advertisements in disguise are inadmissible. 3. In answering a query please quote the number. 4. If a private answer is desired, a stamped addressed envelope must be forwarded.

### Queries.

(11) *Bournemouth.*—Is there a free convalescent home at Bournemouth for males? *Nurse L.*

(12) *Sydney Hospital.*—To whom should I apply in England for a post as nurse in the Sydney Hospital? *A. M. S.*

(13) *Home Wanted.*—"Frigid" would be glad to hear of a home where a nurse could be received permanently. She could not pay more than 7s. a week inclusive.

(14) *Pensions.*—Could a nurse sink a sum of money in the National Pension Fund sufficient to support herself, and if so what sum would be required? *Frigid.*

### Answers.

(11) *Bournemouth.*—No convalescent homes are free. The Firs Home, Bournemouth, is for patients in advanced consumption and the charge is small. The Bournemouth National Sanatorium admits convalescents from consumption, etc., at 7s. 6d. per week. Apply to the secretary, at 28, King-street, St. James's, S.W., which is the London office.

(12) *Sydney Hospital.*—There is no agent in England. The Agent-General for Victoria, Victoria-street, Westminster, might perhaps be able to help you.

(14) *Pensions.*—Unless we are informed of the nurse's age next birthday and the age at which she requires to commence her pension, it is obviously impossible to say what amount will be required to buy a pension. A pension of £10 a year, to commence at the age of 50, can be bought by a nurse aged 23 for £60.

### Appointments.

London Hospital.—Miss Alice Allinson has been appointed night sister; Miss Evelyn Palmer has been appointed sister of the Queen Victoria Wards; Miss Elizabeth Laurie has been appointed sister of the Mary Wards; Miss Anna Louisa Ridal has been appointed sister of the Cotton Wards. All trained at the London Hospital.

### Vacancies.

The following vacancies are announced:

### Matrons.

Monkwearmouth and Southwick Hospital, £35.
South-Western Fever Hospital, £100.

### Night Superintendent.

South Devon Hospital, £30.

### Sisters.

Central London Ophthalmic Hospital.
Royal Albert Hospital.

### Nurses.

Bedford Infirmary, £20.
Birmingham Fever Hospital (several).
Boston Infirmary (several).
Bournemouth Institute, £25.
Bridgwater Infirmary, £25.
Bromwich District Hospital, £22.
Carmarthen Infirmary, £23.
Croydon Institute, £22.
Forest Gate District Schools, £20.
Frome Nurses' Home.
General Nursing Institute.
Hampshire Institute (two), £25.
Heathfield, Norwood.
Hitchin Institution, £23.
Holborn Union, £20.
Jersey Institute, £25.
Kent Nursing Institution.
Kidderminster Infirmary, £20.
King's Cross Hospital, Dundee (two) £24.
Leicester Fever Hospital.
Leith Hospital, N.B., £25.
Mexborough Cottage Hospital, £20.
Newport Infirmary, £25.
Northern Counties Home, Newcastle.
North Riding Infirmary, £20.
Nurses' Home, Newcastle.
Nurses' Home, Ryde.
Nurses' Home, Sheffield.
Nursing Institution, 96, 97, 98, Wimpole-street, London, W., Mr. Wilson has several vacancies for thoroughly trained nurses.
Portsmouth Infectious Hospital.
Scarborough Institute.
Shoreditch Infirmary, £20.
South Devon Hospital.
South London Institute.
Southport Nurses' Home.
St. Helena Home, N.W.
Stockport Infirmary, £25.
St. Peter's, Bedford, £25.
Swansea Institute, £25.
Westhulme Hospital, Oldham, £25.
Wolverhampton Institute.
Workhouse Infirmary Nursing Association.

### District Nurses.

Dulverton, Somerset.
Kemerton Rectory (Midwife).

### Probationers.

Babbacombe Nursery Hospital.
Canterbury Hospital.
Chichester Infirmary.
Hull Royal Infirmary.
Nurses' Home, Sheffield.
Maidstone Hospital.
Ventnor Consumption Hospital.

### Amusements and Relaxation.

**N.B. Third Quarterly Word Competition Commenced October 5th, 1889, ends December 28th, 1889.**

Three Prizes of 15s., 10s., 5s., will be given for the largest number of words derived from the words set for dissection.

Proper names, abbreviations, foreign words, words of less than four letters, and repetitions are barred; plurals, and past and present participles of verbs, are allowed. Nuttall's *Standard* dictionary only to be used.

N.B.—Word dissections must be sent in WEEKLY, arranged alphabetically, with correct total affixed.

The word for dissection for this, the *Fourth* week of the quarter, being "S W A L L O W S."

### Results of Second Week.

| Names. | Oct. 17th. | Totals. | Names. | Oct. 17th. | Totals. |
|---|---|---|---|---|---|
| Winifred | 276 | 294 | Sunshine | 315 | 329 |
| Adelaide | 255 | 273 | Electrician | 363 | 381 |
| Nettie Vance | 284 | 296 | Reldas | 389 | 406 |
| F. W. P. H. | 404 | 421 | H. C. | 227 | 243 |
| Amos | 230 | 247 | Rover | 378 | 395 |
| Reyot | 352 | 369 | Beryl | 177 | 188 |
| A. S. K. | 263 | 275 | Qu'appelle | 338 | 356 |
| Ellice | 438 | 455 | Hercules | — | 12 |
| Neptune | 398 | 415 | Lightowlers | 423 | 439 |
| Ecila | 440 | 457 | Jenny Wren | 311 | 328 |
| Tinie | — | 17 | Oakenrod | 438 | 454 |
| Amicus | 437 | 454 | Gypsye | 368 | 384 |
| Juvenis | 235 | 252 | Leo | 266 | 266 |
| Sister | 424 | 438 | Night Watch | 149 | 149 |
| Jumps | 439 | 456 | Embryo | 205 | 205 |
| M. W. | 418 | 435 | Africando | 158 | 158 |
| Esperance | 418 | 435 | | | |

### Notice to Correspondents.

N.B.—All letters referring to this page which do not arrive at 139 and 140, Salisbury-court, London, E.C., by the *first post on Thursdays*, and are not addressed PRIZE EDITOR, will in future be disqualified and disregarded.

